# A 38-gene model comprised of key *TET2-*associated genes shows additive utility to high-risk prostate cancer cases in the prognostication of biochemical recurrence

**DOI:** 10.1186/s12885-020-07438-4

**Published:** 2020-10-02

**Authors:** Shivani Kamdar, Neil E. Fleshner, Bharati Bapat

**Affiliations:** 1 Lunenfeld-Tanenbaum Research Institute, Mount Sinai Hospital, 60 Murray Street, Toronto, ON M5T 3L9 Canada; 2 Department of Laboratory Medicine and Pathobiology, University of Toronto, Medical Sciences Building (6th floor), 1 King’s College Circle, Toronto, ON M5S 1A8 Canada; 3 Department of Surgery and Surgical Oncology, Division of Urology, University Health Network, University of Toronto, 190 Elizabeth St, Toronto, ON M5G 2C4 Canada

**Keywords:** Prostate cancer, TET2, Gene expression, Statistical models, Biochemical recurrence

## Abstract

**Background:**

Early treatment of patients at risk for developing aggressive prostate cancer is able to delay metastasis and reduce mortality; as such, up-front identification of these patients is critical. Several risk classification systems, including CAPRA-S, are currently used for disease prognostication. However, high-risk patients identified by these systems can still exhibit wide-ranging disease outcomes, leading to overtreatment of some patients in this group.

**Methods:**

The master methylation regulator *TET2* is downregulated in prostate cancer, where its loss is linked to aggressive disease and poor outcome. Using a random forest strategy, we developed a model based on the expression of 38 genes associated with *TET2* utilizing 100 radical prostatectomy samples (training cohort) with a 49% biochemical recurrence rate. This 38-gene model was comprised of both upregulated and downregulated *TET2*-associated genes with a binary outcome, and was further assessed in an independent validation (*n* = 423) dataset for association with biochemical recurrence.

**Results:**

38-gene model status was able to correctly identify patients exhibiting recurrence with 81.4% sensitivity in the validation cohort, and added significant prognostic utility to the high-risk CAPRA-S classification group. Patients considered high-risk by CAPRA-S with negative 38-gene model status exhibited no statistically significant difference in time to recurrence from low-risk CAPRA-S patients, indicating that the expression of *TET2-*associated genes is able to separate truly high-risk cases from those which have a more benign disease course.

**Conclusions:**

The 38-gene model may hold potential in determining which patients would truly benefit from aggressive treatment course, demonstrating a novel role for genes linked to *TET2* in the prognostication of PCa and indicating the importance of *TET2* dysregulation among high-risk patient groups.

## Background

Prostate cancer (PCa) is the most common cancer diagnosed in men worldwide. Overall, PCa has an excellent prognosis, with a 5-year survival rate of 98% [1]. However, PCa is a highly heterogenous disease, with a clinical course that can range from indolent and localized with nearly 100% survival rate to metastatic and lethal. Patients with metastatic disease have a far lower survival rate, ranging between 29 and 31% [2, 3].

Due to this discrepancy in survival rates, early identification of patients at risk of aggressive disease is critical. Early treatment of patients with biochemical recurrence (BCR) by salvage radiation therapy and/or androgen deprivation has been significantly linked to lower incidence of metastasis, and may reduce mortality if administered within 2 years after BCR first occurs; thus, models able to predict BCR risk in the period immediately following surgery are very important for clinical decision-making [4].

Variations of the UCSF Cancer of the Prostate Risk Assessment (CAPRA) score have outperformed other risk classification criteria for prediction of BCR-free survival, with the postsurgical CAPRA-S score exhibiting a slight improvement in c-index for BCR over the presurgical CAPRA score (0.77 compared to 0.69 respectively) in the CaPSURE registry cohort [5–8]. In addition, both scores also show utility in risk analyses for cancer-specific mortality and metastases, making them particularly useful [5, 6, 9].

Multiple genomic models have been shown to add further utility to CAPRA-S risk classification. The Decipher Prostate Cancer Test, which measures the expression levels of 22 genes in radical prostatectomy (RP) specimens, is an independent predictor of metastasis when assessed alongside CAPRA-S, while combination of the Prolaris cell cycle gene-expression test with CAPRA-S results in superior predictive ability for BCR risk [10–12]. As such, continued integration of novel genomic models with CAPRA-S may further improve its prognostication ability. Recently, our group identified a four-gene methylation model which exhibited additive potential to CAPRA-S for association with BCR and prognostication of postsurgical therapies, demonstrating the potential contribution of epigenetic mechanisms in this field as well [13].

In this regard, ten-eleven translocase (*TET*) enzymes, considered master methylation regulators, are aberrantly expressed in multiple cancers. *TET*-mediated regulatory mechanisms present a potentially promising strategy for identifying genes useful in prognostic modeling. Loss of *TET2* expression in particular is correlated with metastasis, increased Gleason score, and worse cancer-specific survival in PCa patients [14–16]. Previously, we used CRISPR-Cas9 directed *TET2* knockout of prostate cells to identify candidate genes whose expression is regulated by *TET2* loss in PCa. Subsequently, we showed that the expression status of seven target genes regulated by *TET2*-mediated promoter methylation is significantly associated with shorter recurrence-free survival time in PCa patients, showing the possible utility of mining both downregulated and upregulated *TET2*-related genes for improved disease prognostication [17].

To further investigate the combinatorial utility of genes associated with *TET2* for improved clinical decision-making, we used a backwards feature selection strategy to generate an optimal 38-gene random forest model with binary outcome, in a training cohort (the Moreno cohort) of 100 formalin-fixed, paraffin-embedded prostate cancer samples [18], and validated this model in the Cancer Genome Atlas (TCGA) prostate tumor dataset [19]. Our 38-gene model (38G) is significantly associated with BCR via Cox regression modeling in both training and validation sets, and exhibits 81.4% sensitivity for BCR in the validation cohort. Most importantly, the 38G model adds significant discriminatory ability to CAPRA-S high-risk cases specifically, as PCa patients with CAPRA-S scores ≥6 and a positive 38G model score exhibit significantly shorter time to BCR than those with negative 38G model scores. Overall, our 38G model is able to differentiate those cases which are truly at an increased risk of progression from those with outcomes similar to CAPRA-S intermediate-risk or low-risk categories, indicating that *TET2*-associated gene dysregulation may be implicated in bona-fide high-risk PCa cases. Further validation of this model in independent cohorts will allow the additive utility of 38G to CAPRA-S risk classification to be more extensively explored.

## Methods

### Patient cohorts

Two publicly available datasets were analysed in this study. The training cohort (Moreno) was comprised of 106 formalin-fixed, paraffin-embedded (FFPE) radical prostatectomy samples from 100 patients, of whom 49 exhibited BCR [18]. The validation cohort (TCGA) was comprised of 423 fresh-frozen radical prostatectomy samples, of whom 43 exhibited BCR [19]. Outcome classification at follow-up was derived from the TCGA database as either exhibiting progressive disease, stable disease, complete remission, or partial remission. BCR was defined as two consecutive postoperative PSA readings ≤0.2 ng/mL. Both cohorts exhibited similar median age and preoperative PSA levels (Table 1). CAPRA-S was calculated on a 12-point scale as per the original system by Cooperberg et al. [6] Briefly, one point was assigned for the presence of extracapsular extension and lymph node invasion, two points for positive surgical margins and seminal vesicle invasion, while Gleason score and PSA were assessed on a point scale from 0 (lowest) to 3 (highest). Patients were assigned to risk categories of CAPRA-S low (0–2 points), intermediate (3–5 points), or high-risk (6–12 points) as per this scale.

**Table 1:** Clinical characteristics of training (Moreno) and validation (TCGA) cohorts

Clinical Characteristic	Moreno Cohort (FFPE)	TCGA Cohort (RP)
Gleason Score	No. of patients (%)	No. of patients (%)
≤ 6 (3 + 3)	11 (11.00%)	37 (8.75%)
7 (3 + 4)	53 (53.00%)	162 (38.30%)
7 (4 + 3)	22 (22.00%)	101 (23.88%)
≥ 8	14 (14.00%)	158 (37.35%)
**Pathological Stage**		
pT2	69 (69.00%)	169 (39.95%)
pT3	2 (2.00%)	0 (0.00%)
pT3a	6 (6.00%)	138 (32.62%)
pT3b	9 (9.00%)	104 (24.59%)
pT4	1 (1.00%)	6 (1.42%)
**Lymph Node Invasion**		
Present	0 (0%)	60 (14.18%)
Absent	37 (37.00%)	331 (78.25%)
**Surgical Margins**		
Positive	39 (39.00%)	116 (27.42%)
Negative	56 (56.00%)	312 (73.76%)
**Age**		
Median	61.7	61
Range	43.0–78.0	41.0–77.0
**Pre-operative PSA (ng/uL)**		
Median	7.2	7.5
Range	1.8–72.6	0.7–107
**Biochemical Recurrence**		
Number of recurrences	49 (49.00%)	43 (10.17%)
Average follow-up time in years (range)	5.79 (0.06–15.26)	3.07 (0.06–13.76)
**Total**	**100**	**423**

### Whole-Transcriptome sequencing and analysis

RNA-sequencing data used in this study was derived from previously published work from our group [17]. Briefly, CRISPR-Cas9 targeting the first coding exon of *TET2* was used to achieve *TET2* knockout in normal prostate (RWPE-1) cells. Whole RNA extracted via TRIzol was sequenced and aligned at The Centre for Applied Genomics (TCAG, Toronto).

### Gene selection

An initial list of genes was identified based on the following characteristics: firstly, genes exhibiting significant gain of expression (> 1.5-fold increase, *p* < 0.05) or loss of expression (> 1.5-fold decrease, *p* < 0.05) in *TET2-*knockout cells as compared to unmodified parental RWPE-1 cells by edgeR; secondly, genes exhibiting significantly increased or decreased expression respectively in a low-*TET2* expressing subset (bottom 10th percentile for *TET2* expression) of the TCGA dataset as compared to the remaining tumors (*p* < 4.46E-5, Mann-Whitney U test). An expanded description of selection criteria is presented in the Supplementary Methods section. The intersection of these gene lists was used to form a final gene set of 1122 genes. These were considered high-confidence TET2-associated genes, and were used for downstream model generation analyses.

### Model generation and optimization

We used random forest-based recursive feature elimination using bootstrapping as the resampling method (*n* = 75), with the number of selected features set from 2 to 50, to select a final list of genes from the high-confidence *TET2*-associated genes identified in the previous step. The random forest model was trained on the selected features, using 10-fold cross-validation to optimize the number of variables available for splitting at each node, tree size, and tree depth. All model training was performed in the Moreno cohort, and validation was performed using the TCGA cohort. All random forest analyses used biochemical recurrence as the outcome, and were performed using the caret package of R (v6.0.84).

### Statistical analyses

Association between candidate gene expression and tumor versus normal status was analyzed using Mann-Whitney U tests as part of the base “stats” package of R. Bonferroni correction was applied by dividing 0.05 by the number of samples analyzed and using the resultant value as the confidence threshold. Sensitivity, specificity, positive predictive value, and negative predictive value for BCR at various timepoints were calculated using the confusionMatrix function from the caret package of R. Univariate and multivariate Cox regression analyses, as well as Kaplan-Meier survival curves, were performed using the survival package of R, using log-rank *p*-values to determine significance.

We used *p* < 0.05 as the confidence threshold for the above analyses unless otherwise specified in the manuscript. All statistical analyses were performed using R (v3.6.1).

## Results

### Optimal gene model selection by random forest

High-confidence *TET2-*associated genes exhibiting expression changes in PCa were identified as previously described [17], and were assessed by random forest modeling in the training cohort to determine which of these genes would provide the best discriminatory power for prognostication of biochemical recurrence (BCR). As the Moreno cohort had a BCR rate of 49%, it was chosen as the training cohort to ensure an equal distribution of cases for model optimization (Supplementary Figure 1).

Of the 1122 high-confidence *TET2*-associated genes identified, backwards feature selection-based random forest modeling chose a 38-gene model which consisted of 18 upregulated and 20 downregulated genes in PCa (Supplementary Table 1, Supplementary Figures. 2–3). The 38G model was optimized for predictive accuracy in the training cohort, and was designed to favour correct classification of positive cases. This 38G model had a negative predictive value (NPV) of 94.12%, sensitivity of 94.4, and log-rank *p*-value <2E-16 for prediction of overall BCR within a follow-up period of 14.26 years in the training cohort (Table 2). The 38G model also exhibited 100% NPV for prediction of early BCR within a 1.5-year period post RP. In comparison, CAPRA-S exhibited lower sensitivity (37% versus 94%), slightly higher specificity (94.0% versus 92.31%) and slightly lower positive predictive value (PPV; 86.96% versus 92.73%) over the same time period for overall BCR when using a high-risk CAPRA-S score (≥6) as the cutoff for binary dichotomization (Table 2).

**Table 2:** 38G model performance compared to CAPRA-S for association with BCR in the training cohort (*n* = 100)

	Sensitivity	Specificity	PPV	NPV
*Overall BCR*				
**38G**	94.44	92.31	92.73	94.12
**CAPRA-S**	37.74	94	86.96	58.75
*BCR within 1.5 years*				
**38G**	100	62.65	42.59	100
**CAPRA-S**	52.17	86.25	52.17	86.25
*BCR within 3 years*				
**38G**	95.35	79.37	75.93	96.15
**CAPRA-S**	41.86	91.67	78.26	68.75
*BCR within 5 years*				
**38G**	94.12	89.09	88.89	94.23
**CAPRA-S**	40	94.34	86.96	62.5
*BCR within 7 years*				
**38G**	92.31	88.89	88.89	92.31
**CAPRA-S**	39.22	94.23	86.96	61.25

### Validation of the 38G model for prognostication of BCR

**Fig. 1:**
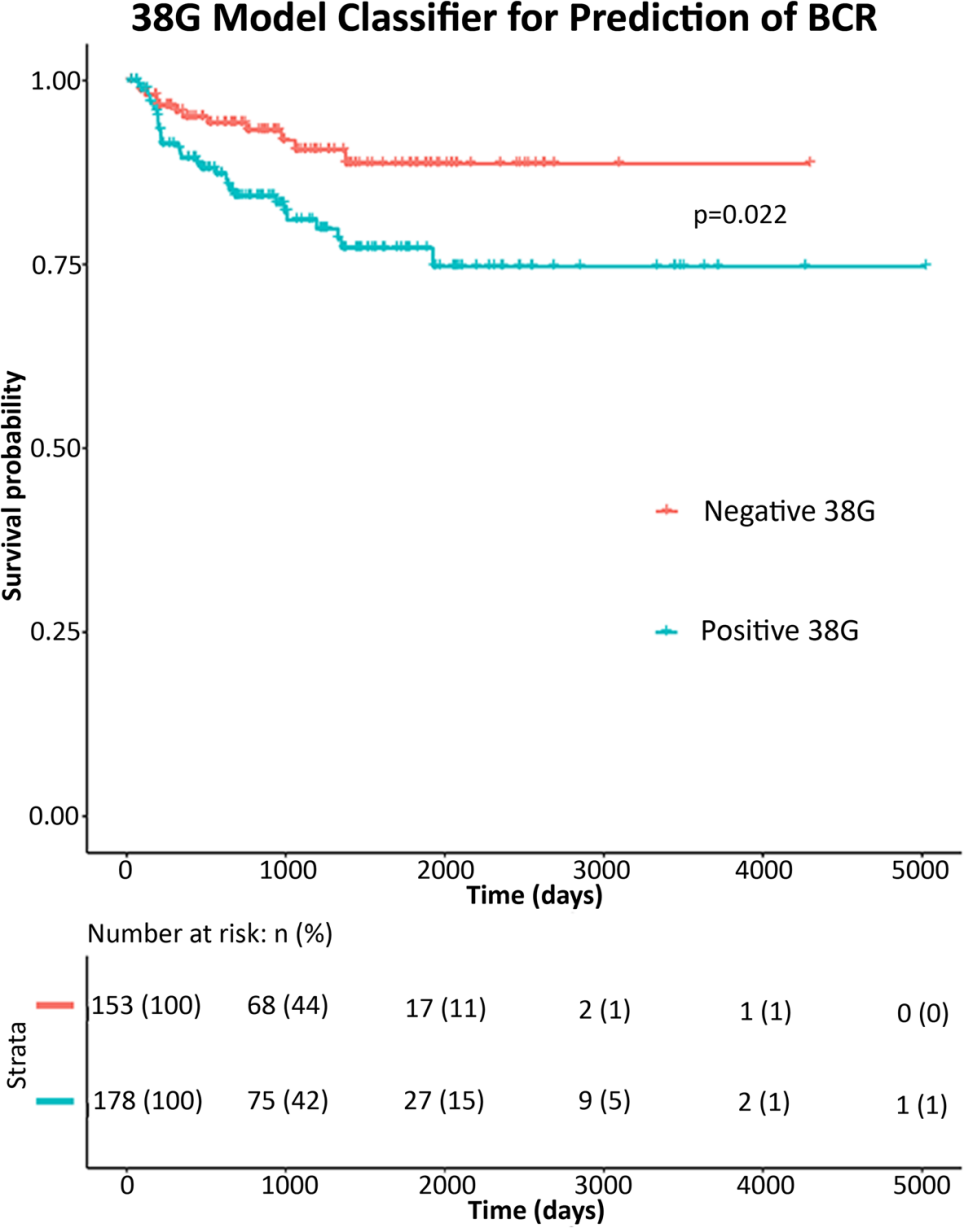
Univariate Kaplan-Meier curve for prediction of BCR in the validation (TCGA) cohort, comparing survival probability between negative (0) or positive (1) 38G model result, with log-rank *p*-value outlined. *Below:* Risk table indicating the number of patients in each category at risk at various timepoints. Figure generated using the R (v3.6.1) package survminer (v0.4.6)

The TCGA cohort was used for validation of the prognostic accuracy of the 38G model, which exhibited an NPV of 93.39% and sensitivity of 81.4% for prediction of overall BCR within a follow-up period of 13.76 years in the validation cohort (Table 3). On univariate Cox proportional hazards analysis, binary 38G model score was significant for association with BCR, with a positive model score giving a 2.46-fold increased risk of a patient exhibiting BCR (95%CI 1.14–5.3; log-rank *p*-value 0.022). (Fig. 1) When compared to CAPRA-S in the same cohort, our model outperformed CAPRA-S in terms of sensitivity for overall BCR and at timepoints of BCR within 1.5, 3, 5, or 7 years; CAPRA-S favored higher specificity and PPV for the same timepoints (Table 3).

**Table 3:** 38G model performance compared to CAPRA-S for association with BCR in the validation cohort (*n* = 423)

	Sensitivity	Specificity	PPV	NPV
*Overall BCR*				
**38G**	81.4	36.33	15.02	93.39
**CAPRA-S**	52.5	72.67	20.39	91.98
*BCR within 1.5 years*				
**38G**	77.78	35.17	9.01	95.04
**CAPRA-S**	52	71.43	12.62	94.94
*BCR within 3 years*				
**38G**	81.58	36.08	13.3	89.27
**CAPRA-S**	51.43	72.13	17.48	92.83
*BCR within 5 years*				
**38G**	80.95	36.22	14.59	93.39
**CAPRA-S**	53.85	72.76	20.39	92.41
*BCR within 7 years*				
**38G**	81.4	36.33	15.02	93.39
**CAPRA-S**	52.5	72.67	20.39	91.98

In order to determine whether our model was an independent predictor of BCR in this cohort, we performed multivariate Cox regression analysis, evaluating our 38G model as compared to CAPRA-S scores categorized into low-risk (score ≤ 2), intermediate-risk (score 3–5), or high-risk (score ≥ 6) classification groups (Table 4, Supplementary Figure 4).

**Table 4:** Univariate and multivariate Cox regression analyses for 38G and CAPRA-S in the validation (TCGA) cohort

	Hazard ratio	2.50%	97.50%	log-rank ***p***-value
*Univariate*				
**38G**	2.458	1.14	5.3	0.0218
**CAPRA-S**	2.209	1.441	3.387	2.77E-04
*Multivariate (low* vs *intermediate* vs *high risk CAPRA-S)*				
**38G**	2.222	0.976	5.059	0.0571
**CAPRA-S**	2.073	1.344	3.198	9.81E-04
*Multivariate (high-risk* vs. *low/intermediate-risk CAPRA-S)*				
**38G**	2.799	1.498	5.229	0.0013
**CAPRA-S**	2.362	1.039	5.37	4.03E-02

*CAPRA-S has been assessed as per categorical risk classification: low, intermediate, and high-risk*

*Multivariate analysis 1: HR represents increased risk in intermediate-risk cases as compared to low-risk, or in high-risk cases as compared to intermediate-risk*

*Multivariate analysis 2: HR represents increased risk in high-risk cases as compared to low- or intermediate-risk cases*

Although our model was not an independent predictor of BCR when combined with CAPRA-S risk categories, its trending *p*-value (*p* = 0.057) indicated that it may possess some additive utility to one or more risk categories in particular.

### The 38G model adds significant prognostic utility to CAPRA-S high-risk classification patients

We examined the additive potential of the 38G model by assessing it in combination with CAPRA-S risk categories via Kaplan-Meier analysis. On its own, there was no significant difference in outcome between CAPRA-S low- and intermediate-risk groups in the TCGA cohort; however, both groups exhibited significantly longer time to progression than those in the high-risk group (Supplementary Figure 5).

**Fig. 2:**
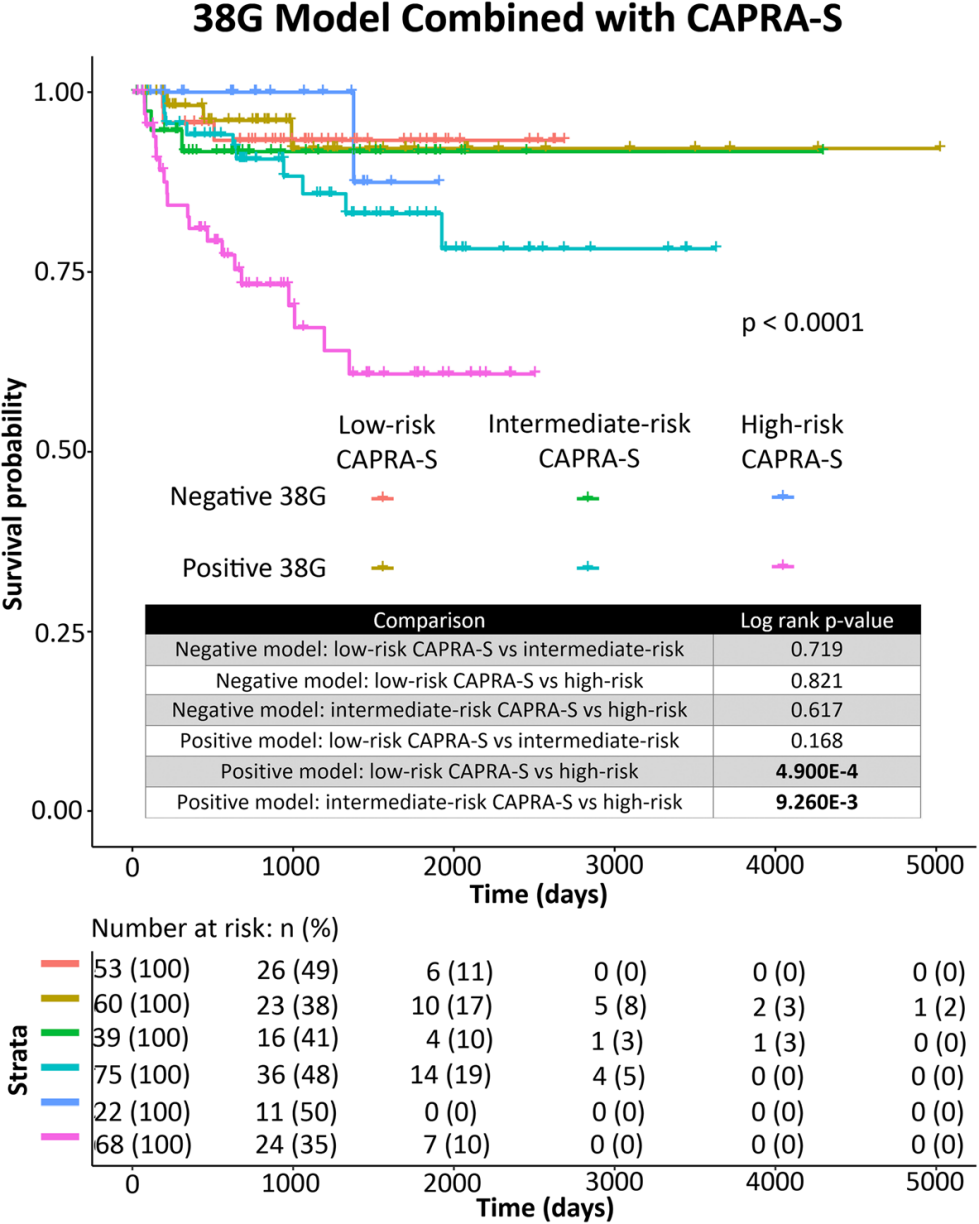
Multivariate Kaplan-Meier curve for prediction of BCR in the validation (TCGA) cohort. Binary 38G model is assessed alongside CAPRA-S divided into three categories: low-risk, intermediate-risk, or high-risk. Log-rank *p*-values for pairwise comparisons among negative and positive model subsets are indicated in the accompanying chart. Overall log-rank *p*-value is indicated on the graph. *Below:* Risk table indicating the number of patients in each group at risk at various timepoints. Figure generated using the R (v3.6.1) package survminer (v0.4.6)

**Fig. 3:**
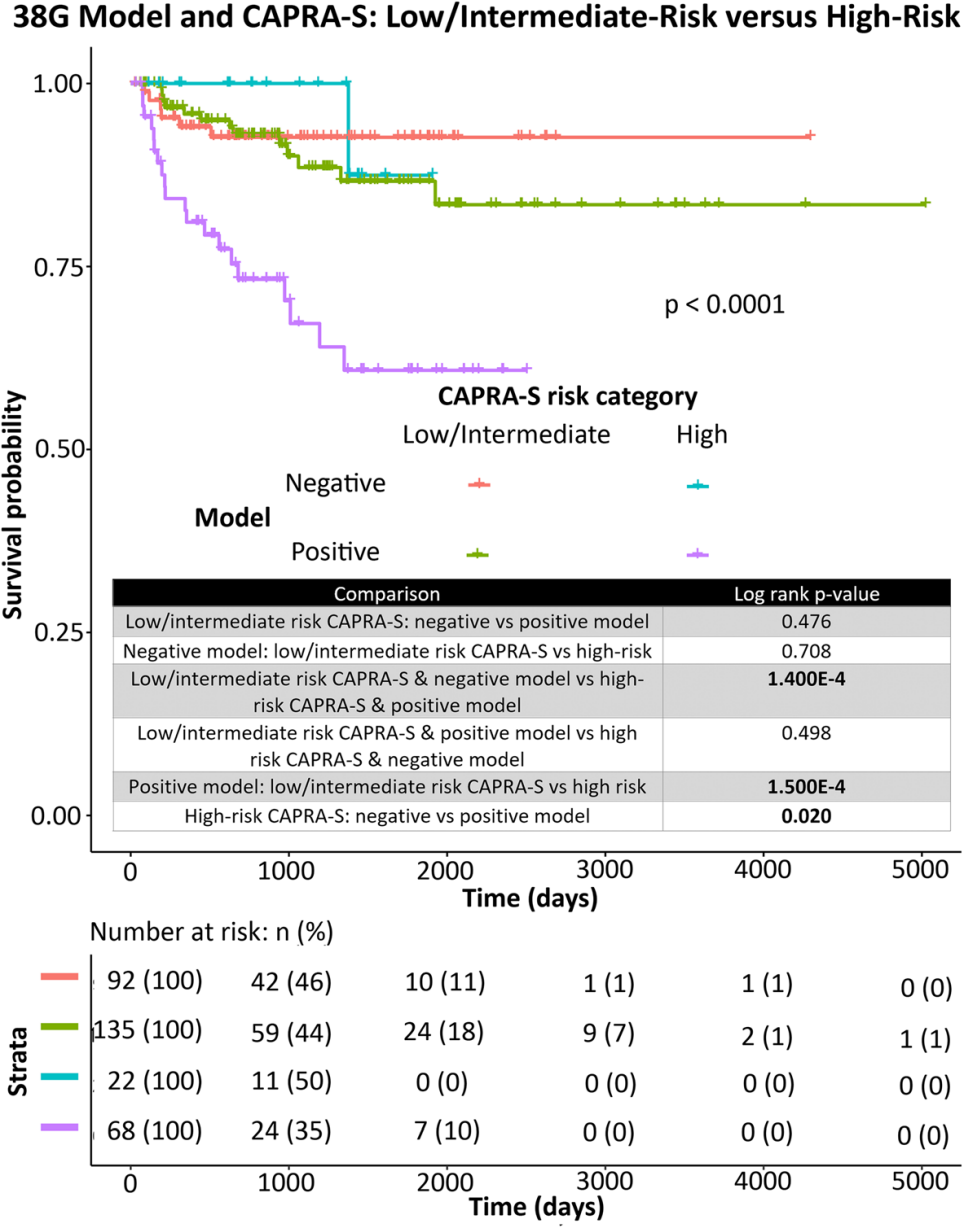
Multivariate Kaplan-Meier curve for prediction of BCR in the validation (TCGA) cohort. Binary 38G model is assessed alongside CAPRA-S divided into two categories: low-risk/intermediate risk, or high-risk. Log-rank *p*-values for pairwise comparisons are indicated in the accompanying chart. Overall log-rank *p*-value is indicated on the graph. *Below:* Risk table indicating the number of patients in each group at risk at various timepoints. Figure generated using the R (v3.6.1) package survminer (v0.4.6)

**Fig. 4:**
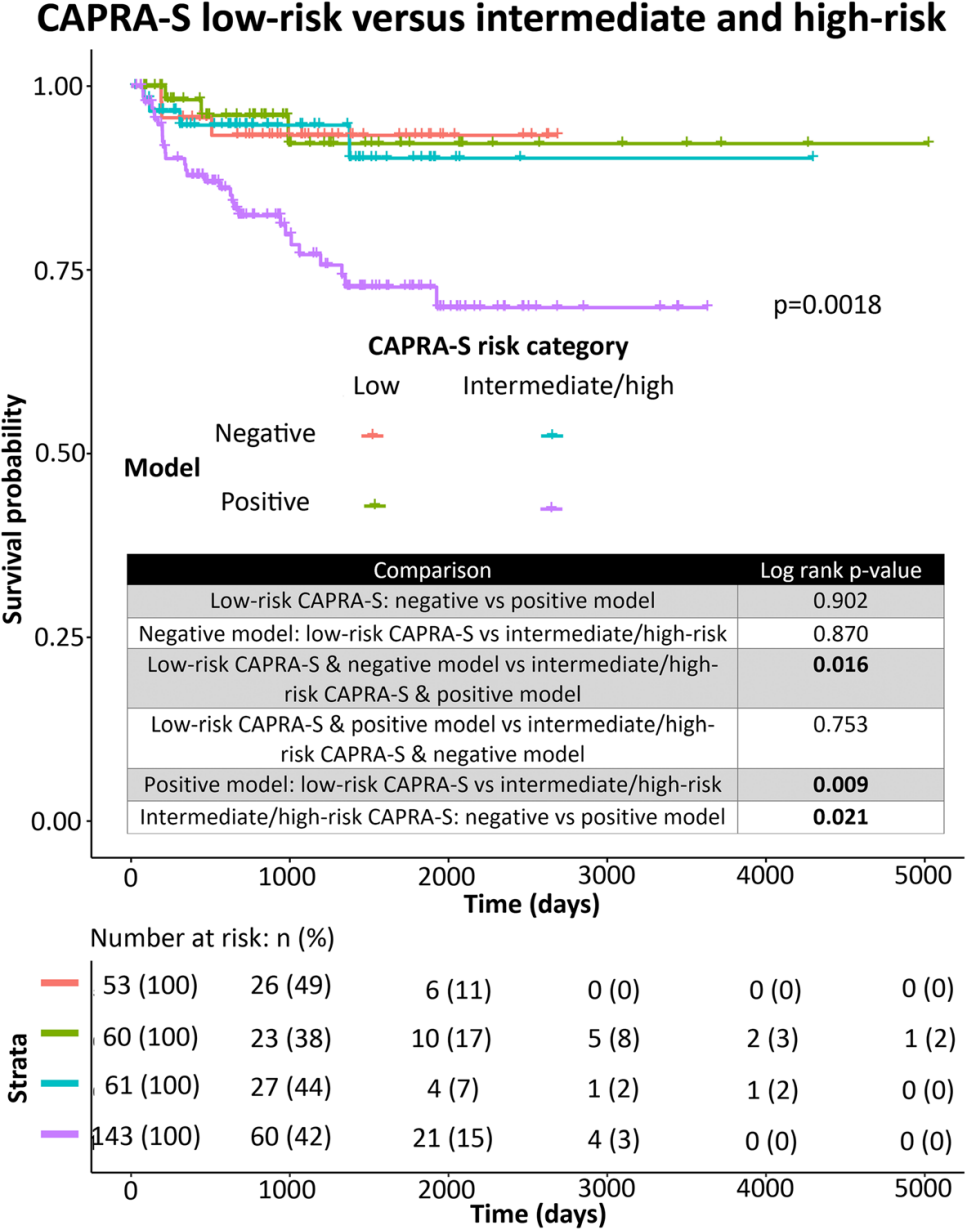
Multivariate Kaplan-Meier curve for prediction of BCR in the validation (TCGA) cohort. Binary 38G model is assessed alongside CAPRA-S divided into two categories: low-risk, or intermediate/high-risk. Log-rank *p*-values for pairwise comparisons are indicated in the accompanying chart. Overall log-rank *p*-value is indicated on the graph. *Below:* Risk table indicating the number of patients in each group at risk at various timepoints. Figure generated using the R (v3.6.1) package survminer (v0.4.6)

Intriguingly, addition of our random forest classifier to high-risk CAPRA-S was able to significantly improve prognostication of BCR. Among high-risk CAPRA-S cases, 38G-positive cases exhibited significantly worse outcome than 38G-negative cases. In multivariate analysis, the 38G model was an independent predictor when combined with binary CAPRA-S high-risk versus CAPRA-S intermediate-or low-risk classifications (Table 4) and improved the c-index of CAPRA-S alone from 0.660 to 0.680. Furthermore, despite the fact that high-risk CAPRA-S cases exhibited significantly worse outcome in terms of BCR than intermediate- or low-risk cases when assessed alone, high-risk cases did not significantly differ from intermediate-risk and/or low-risk cases when the 38G classifier was negative (Figs. 2, 3, 4).

Overall, these results indicate that a negative 38G result is strongly indicative of better outcome in terms of BCR. As such, a positive 38G result is able to distinguish those cases which are truly high-risk for BCR from those which exhibit similar outcomes to intermediate- or even low-risk CAPRA-S cases, adding significant utility to CAPRA-S risk classification.

The 38G model did not add significant discriminatory ability to CAPRA-S low-risk or CAPRA-S intermediate-risk cases alone (Fig. 2).

### 38G model classifier is an independent predictor of tumor outcome in patients

We next examined the association of 38G classifier status with tumor outcome on patient follow-up in the validation dataset. On its own, a positive 38G model result was significantly associated with increased likelihood of a patient exhibiting partial remission or progressive disease as compared to complete remission (OR: 7.99, 95% CI 2.28–50.66, *p* = 5.65E-3). When combined with categorical CAPRA-S, the 38G model remained an independent predictor of partial remission or progressive disease (OR: 5.70, 95% CI 1.57–36.66, *p* = 2.26E-2), indicating that the 38G classifier is significantly associated with patient outcome (Table 5).

**Table 5:** Logistic regression analyses for 38G and CAPRA-S for association with patient outcome (TCGA cohort)

	Odds ratio	2.50%	97.50%	***p***-value
*Univariate*				
**38G**	7.99	2.28	50.66	5.65E-03
**CAPRA-S**	2.99	1.68	5.72	4.18E-04
*Multivariate*				
**38G**	5.7	1.57	36.66	2.26E-02
**CAPRA-S**	2.5	1.39	4.85	3.65E-03

*Note: CAPRA-S has been assessed as per categorical risk classification: low, intermediate, and high-risk*

## Discussion

Although statistically significant differences in BCR rates between the three different CAPRA-S risk groups have been validated in multiple cohorts [5–8, 20, 21], heterogeneity of clinical outcome is still observed among the high-risk CAPRA-S group. As such, while some patients with high-risk CAPRA-S scores may benefit from multimodal therapy up-front, a proportion of these high-risk patients will not go on to develop BCR or metastasis, and will experience unnecessary morbidity from early treatment [22]. Genomic markers, either individually (such as the ability of *SPINK1* overexpression to significantly predict BCR independently of pathological features) or in combination (in models such as Decipher or Prolaris), have shown utility in distinguishing patients at risk of BCR from those at low risk of progression.

Our 38G model, when combined with CAPRA-S risk classification categories, is able to distinguish patients who are truly at high risk of BCR and should receive timely multimodal therapy from those whose risk of BCR does not actually differ from CAPRA-S low-risk patients, and may benefit from active surveillance programs instead. Furthermore, 38G model status is an independent predictor of patient outcome in the TCGA cohort, and may hold potential as a further indicator of which patients would most benefit from early treatment. These findings suggest that differential expression of *TET2-*associated genes may affect disease progression. Overall, this model integrates both genomic expression data and epigenomic regulation by selecting candidate genes governed by *TET2* in prostate cancer.

Currently available genomic models which have been used to prognosticate recurrence from RP samples include Decipher and Prolaris, which use continuous classifier scores for prediction. Although Decipher is most commonly used for prognostication of metastasis or PCa-specific mortality, one study showed that it improved c-index for prediction of BCR from 0.64 to 0.69 when added to continuous CAPRA-S scores [23]. In contrast, multiple studies have assessed the ability of Prolaris to predict BCR, with univariate HRs ranging from 1.44 to 1.89 across both biopsy and RP specimens [24–26]. In comparison, our binary model exhibits a more modest c-index improvement of 0.02 in the validation cohort, with a univariate HR of 2.46 for BCR. As a closer comparison, the recently published binary copy number-based GEMCaP signature exhibited an HR of 2.69 in a cohort of 140 PCa patients, and was shown on multivariate analysis of categorial CAPRA-S risk groups to provide significant (*p* = 0.012) additive utility to intermediate-risk CAPRA-S cases only, while our model added significantly to high-risk CAPRA-S cases specifically [27]. These studies highlight the differing potential contributions of both genomic and epigenomic mechanisms to different PCa risk groups and outcomes in disease.

Aberrant androgen receptor activation, the key driver of PCa development, represses *TET2* both directly via enhancer binding and indirectly via induction of inhibitory miRNAs 29a and 29b, indicating an important and specific role for *TET2* loss in PCa [15, 16]. However, until recently, the role of specific *TET2*-associated genes in disease progression was relatively unexplored. Here, we have demonstrated the combinatorial efficacy of genes associated with *TET2* in improved prognostication of PCa.

Our previous studies identified seven genes governed by *TET2-*mediated methylation and significantly associated with recurrence in the TCGA dataset, which were among the 1122 *TET2-*associated genes used as the base gene set to generate the 38G model. However, none of these genes were among the final candidates selected as part of the model. Due to the recursive feature elimination method used to generate the model, other genes within the training (Moreno) dataset may have been determined to have greater importance to a combinatorial model, even though these seven genes were found to be significant individually in the TCGA dataset [17]. Furthermore, expression of these genes may also have been correlated with that of other genes, resulting in their feature importance being decreased by random forest modeling.

The thirty-eight upregulated or downregulated *TET2*-linked genes comprising our model have a variety of functions, and are enriched via pathway analysis for lipid binding and transport, oxidoreductase and transferase activity, and cholesterol or steroid esterification, reflecting the importance of steroid metabolism in PCa development and progression. Among these genes, several have been identified as known oncogenes or tumor suppressors in prostate or other cancers. For example, tyrosine kinase non receptor 2 *(TNK2)* promotes androgen receptor transcription and is a critical oncogene in castration-resistant prostate cancer [28], while the retinoic acid synthesis enzyme aldehyde dehydrogenase 1 family member A2 *(ALDH1A2)* is a known candidate tumor suppressor associated with decreased colony formation in PCa cell lines [29]. Several other genes in our model have been independently verified as oncogenes (*SPAG5, PARM1)* or tumor suppressors *(VEPH1, GLCE)* in prostate or other cancers, showcasing the ability of our *TET2*-based model to capture these key changes [30–35]. Our work shows, for the first time, the regulation of these genes by *TET2* loss, which may constitute a novel epigenetic mechanism contributing to the expression changes exhibited by these candidates in PCa.

There are some limitations to this study. A major advantage of the CAPRA-S score is its utility in predicting metastasis and cancer-specific survival outcomes. However, as there were very few cases in the TCGA cohort which exhibited either metastasis or cancer-specific death, the ability of the model to add to CAPRA-S for prediction of these outcomes could not be assessed. Furthermore, in accordance with our previously published strategy for candidate gene identification, the TCGA cohort was initially used to identify high confidence *TET2* associated genes. Although this analysis was independent of gene association with BCR, these findings should be further validated in independent testing cohorts in future studies in order to confirm the potential of the 38G model. An advantage of our model generation strategy using the random forest approach in identifying risk models for PCa recurrence is that the prostate cancer datasets used in this study may also be examined independent of *TET2-*related parameters, or using differing selection criteria, to generate and characterize novel risk gene models using a similar method. Finally, as *TET2-*associated gene expression at RP defines a subset of cases with significantly worse prognosis in the tested cohorts, the biological role of *TET2* in high-risk PCa could also be examined further through in vitro studies to determine whether knockdown or induction of these genes is associated with motility or proliferation in prostate cancer cells.

## Conclusions

Distinguishing prostate cancer patients at high risk for recurrence from those at low risk up-front is an important step influencing clinical decision-making for patient treatment. Our results show the additive potential of an expression-based 38G model, comprised of genes associated with *TET2*, to high-risk CAPRA-S classification for further delineation and accurate /refined prediction of BCR. In future studies, validation of the 38G model alongside CAPRA-S in additional cohorts with expanded information on other disease outcomes will allow the predictive ability of our gene model to be confirmed, and may be able to further elucidate the link between *TET2-*associated genes and high-risk outcomes in prostate cancer.

## Supplementary information

**Additional file 1: Supplementary Figure 1.** Unsupervised heatmap depicting FPKM-normalized expression values in the training (Moreno) cohort (*n* = 100) for the 1122 *TET2*-associated genes identified in this study. Expression gradient bar indicates log10-transformed expression levels, ranging from highest (pale yellow) to lowest (black). Dendrograms indicate clustering between genes (top) or tissue samples (left). Figure generated using the R (v3.6.1) packages viridis (v0.5.1) and pheatmap (1.0.12).

**Additional file 2: Supplementary Figure 2.** Unsupervised heatmap depicting FPKM-normalized expression values in the training (Moreno) cohort (*n* = 100) for the 38 genes comprising our model. Expression gradient bar indicates log10-transformed expression levels, ranging from highest (pale yellow) to lowest (black). Dendrograms indicate clustering between genes (top) or tissue samples (left). Figure generated using the R (v3.6.1) packages viridis (v0.5.1) and pheatmap (1.0.12).

**Additional file 3: Supplementary Figure 3.** Forest plot depicting individual hazard ratios for each of the 38 genes comprising our model, generated using the ggforest function of the survminer (v0.4.6) package of R (v3.6.1). log-rank *p*-values are listed on the right, with statistical significance indicated by asterisks: *0.01 < *p* ≤ 0.05; **0.001 < *p* ≤ 0.01; ***p ≤ 0.001

**Additional file 4: Supplementary Figure 4.** Density histogram plots for 38G-positive and 38G-negative cases within the validation (TCGA) cohort, stratified by (A) continuous CAPRA-S risk score, or (B) CAPRA-S risk category, divided into low (0–2), intermediate (3–5), or high (6–12) risk groups. Overlaid density plots in (A) highlight the differences in peaks and distribution between gene model-selected and gene model-negative cases among the CAPRA-S risk scores. Figure generated using the R (v3.6.1) package ggplot2 (v3.2.1).

**Additional file 5: Supplementary Figure 5.** Univariate Kaplan-Meier curve for prediction of BCR in the validation (TCGA) cohort. The three risk categories of CAPRA-S are assessed, with log-rank p-values for pairwise comparisons between risk categories indicated in the accompanying chart. Overall log-rank p-value is indicated on the graph. *Below:* Risk table indicating the number of patients in each group at risk at various timepoints. Figure generated using the R (v3.6.1) package survminer (v0.4.6).

**Additional file 6. Supplementary Table 1.**

**Additional file 7. Supplementary Methods.**

## Data Availability

The datasets analysed during the current study are available in the following repositories: CRISPR-*TET2-*knockout cell line data is available in the Gene Expression Omnibus (GEO) repository under accession number GSE128399, https://www.ncbi.nlm.nih.gov/geo/query/acc.cgi?acc=GSE128399 [17]. The publicly available Moreno FFPE prostate cancer data set is available in the GEO repository under accession number GSE54460, https://www.ncbi.nlm.nih.gov/geo/query/acc.cgi [18]. The Cancer Genome Atlas datasets analysed in this study are publicly available online at https://portal.gdc.cancer.gov. [19]

## Abbreviations

38G: 38-Gene model
BCR: Biochemical recurrence
CAPRA-S: Postsurgical UCSF cancer of the prostate risk assessment
FFPE: Formalin-fixed, paraffin-embedded
NPV: Negative predictive value
OR: Odds ratio
PCa: Prostate cancer
PPV: Positive predictive value
RP: Radical prostatectomy
