# Supplementary figures and images for: A 38-gene model comprised of key TET2-associated genes shows additive utility to high-risk prostate cancer cases in the prognostication of biochemical recurrence
Source: BMC Cancer. 2020 Oct 2;20:953. doi: 10.1186/s12885-020-07438-4 (PMC7530956; doi:10.1186/s12885-020-07438-4)

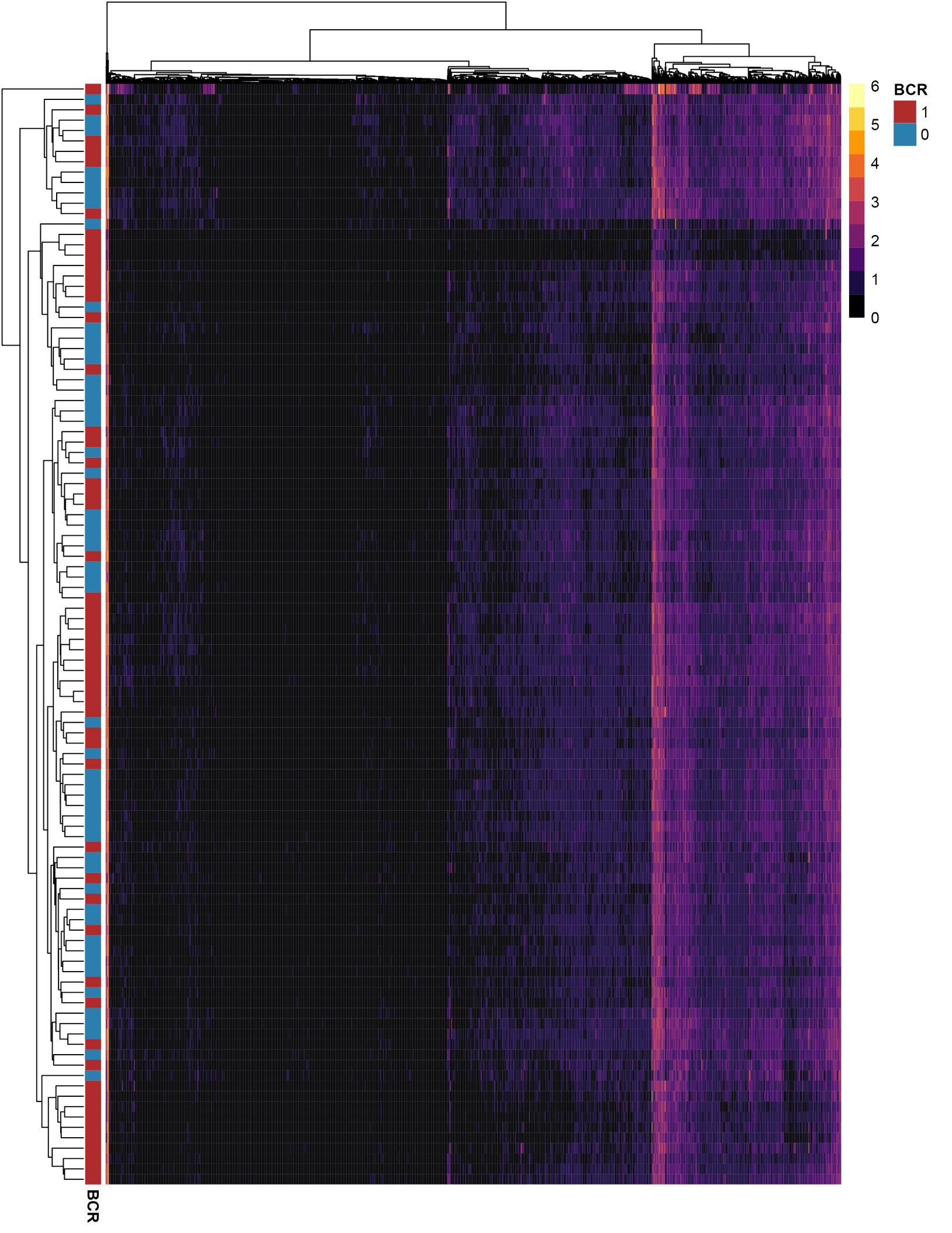

Supplement: Supplementary file 1 — Additional file 1: Supplementary Figure 1. Unsupervised heatmap depicting FPKM-normalized expression values in the training (Moreno) cohort (n = 100) for the 1122 TET2-associated genes identified in this study. Expression gradient bar indicates log10-transformed expression levels, ranging from highest (pale yellow) to lowest (black). Dendrograms indicate clustering between genes (top) or tissue samples (left). Figure generated using the R (v3.6.1) packages viridis (v0.5.1) and pheatmap (1.0.12). [file 12885_2020_7438_MOESM1_ESM.pdf]

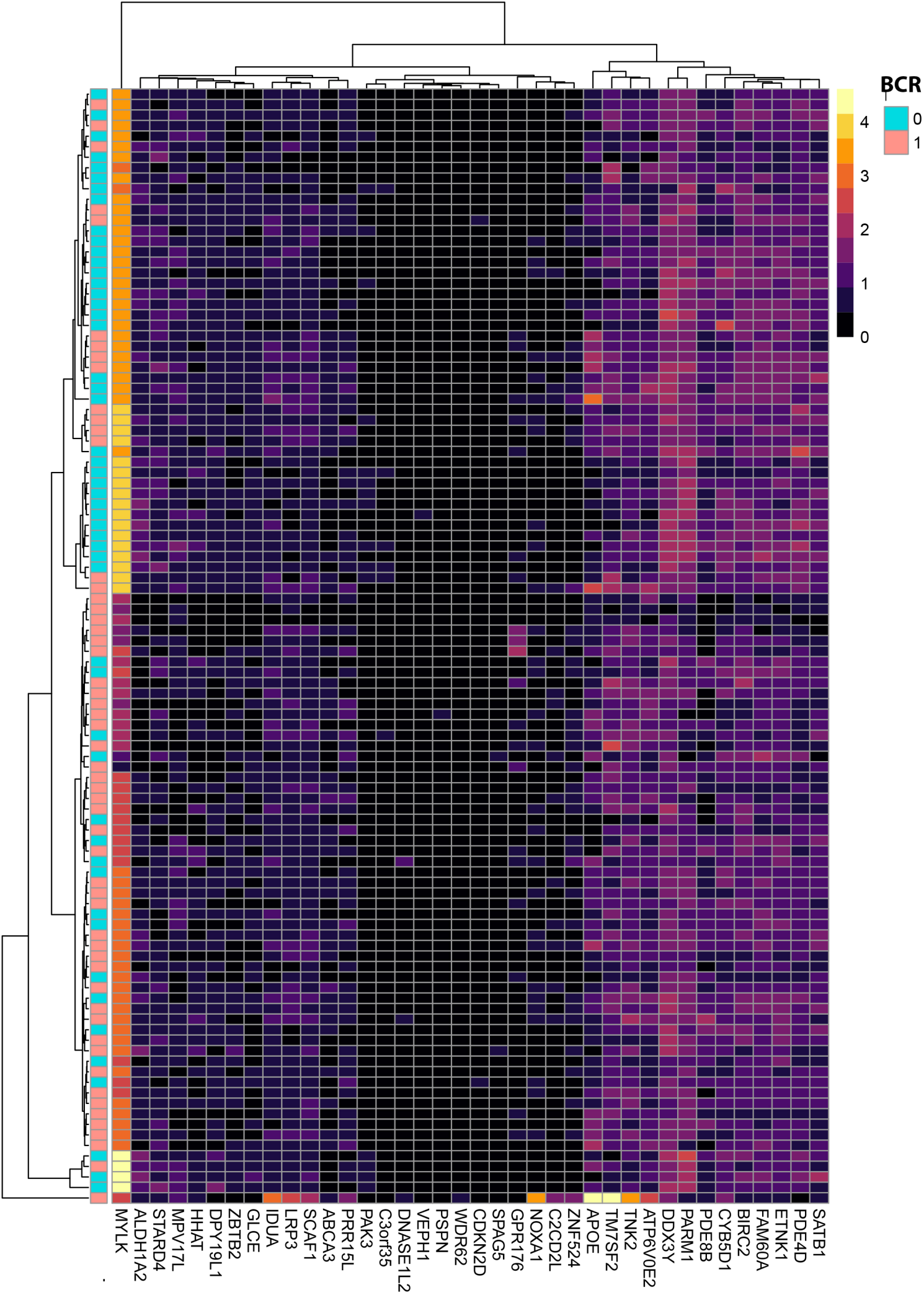

Supplement: Supplementary file 2 — Additional file 2: Supplementary Figure 2. Unsupervised heatmap depicting FPKM-normalized expression values in the training (Moreno) cohort (n = 100) for the 38 genes comprising our model. Expression gradient bar indicates log10-transformed expression levels, ranging from highest (pale yellow) to lowest (black). Dendrograms indicate clustering between genes (top) or tissue samples (left). Figure generated using the R (v3.6.1) packages viridis (v0.5.1) and pheatmap (1.0.12). [file 12885_2020_7438_MOESM2_ESM.pdf]

# Hazard ratio

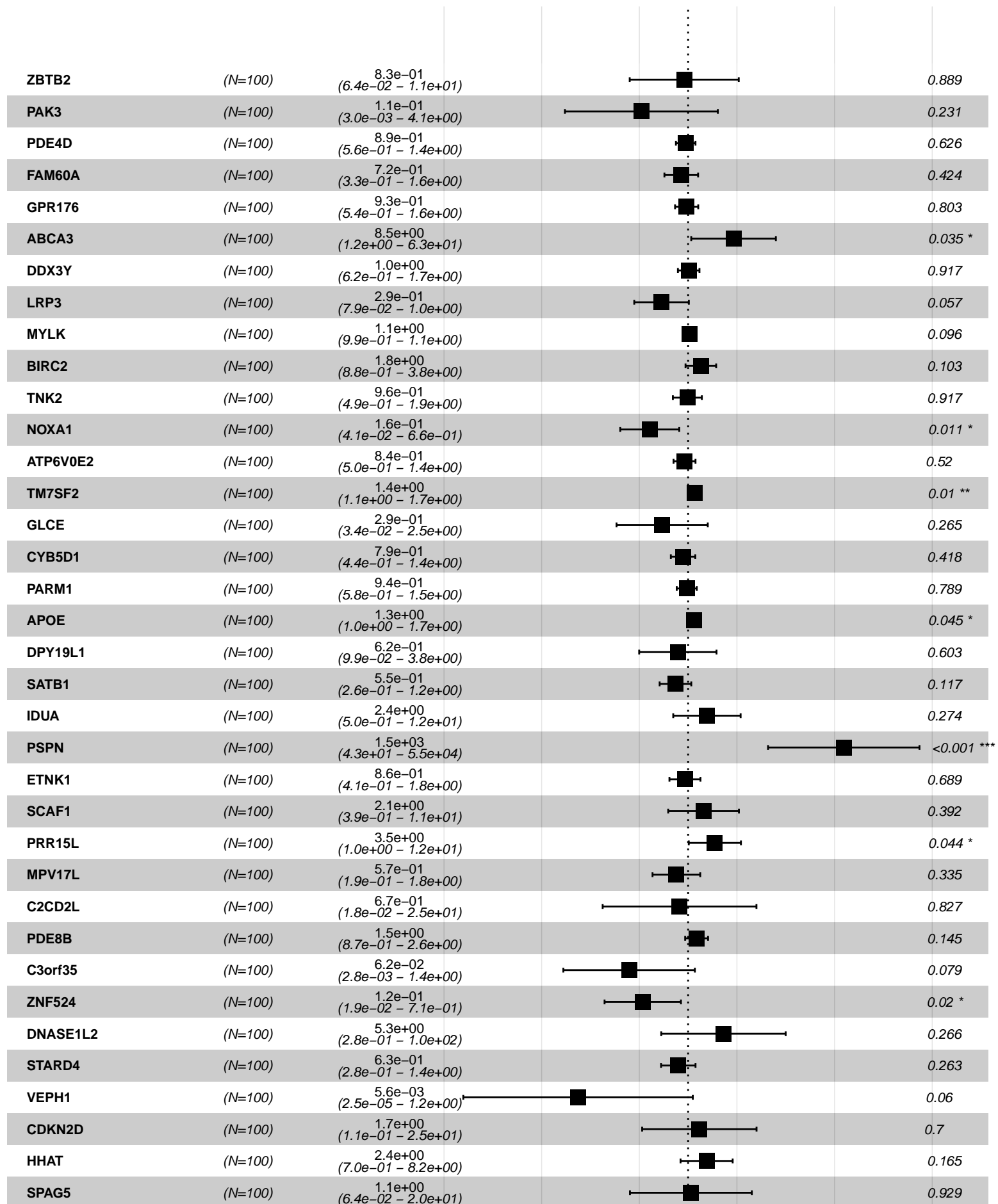

# Events: 49; Global p-value (Log-Rank): 2.0197e-06

AIC: 392.87; Concordance Index: 0.85

1e-05

0.001

0.1

10

1000

100000

Supplement: Supplementary file 3 — Additional file 3: Supplementary Figure 3. Forest plot depicting individual hazard ratios for each of the 38 genes comprising our model, generated using the ggforest function of the survminer (v0.4.6) package of R (v3.6.1). log-rank p-values are listed on the right, with statistical significance indicated by asterisks: *0.01 < p ≤ 0.05; **0.001 < p ≤ 0.01; ***p ≤ 0.001 [file 12885_2020_7438_MOESM3_ESM.pdf]

**A**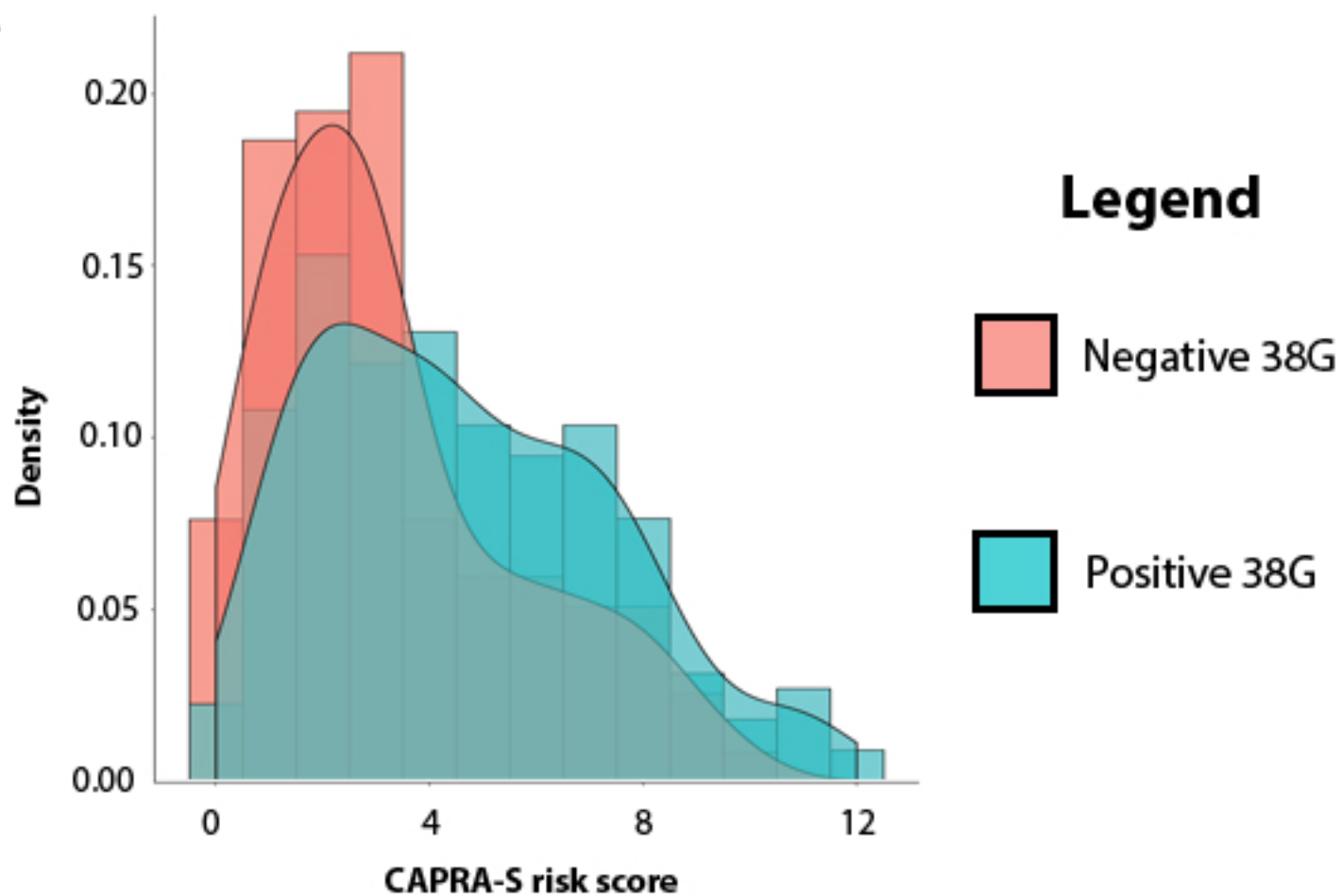**B**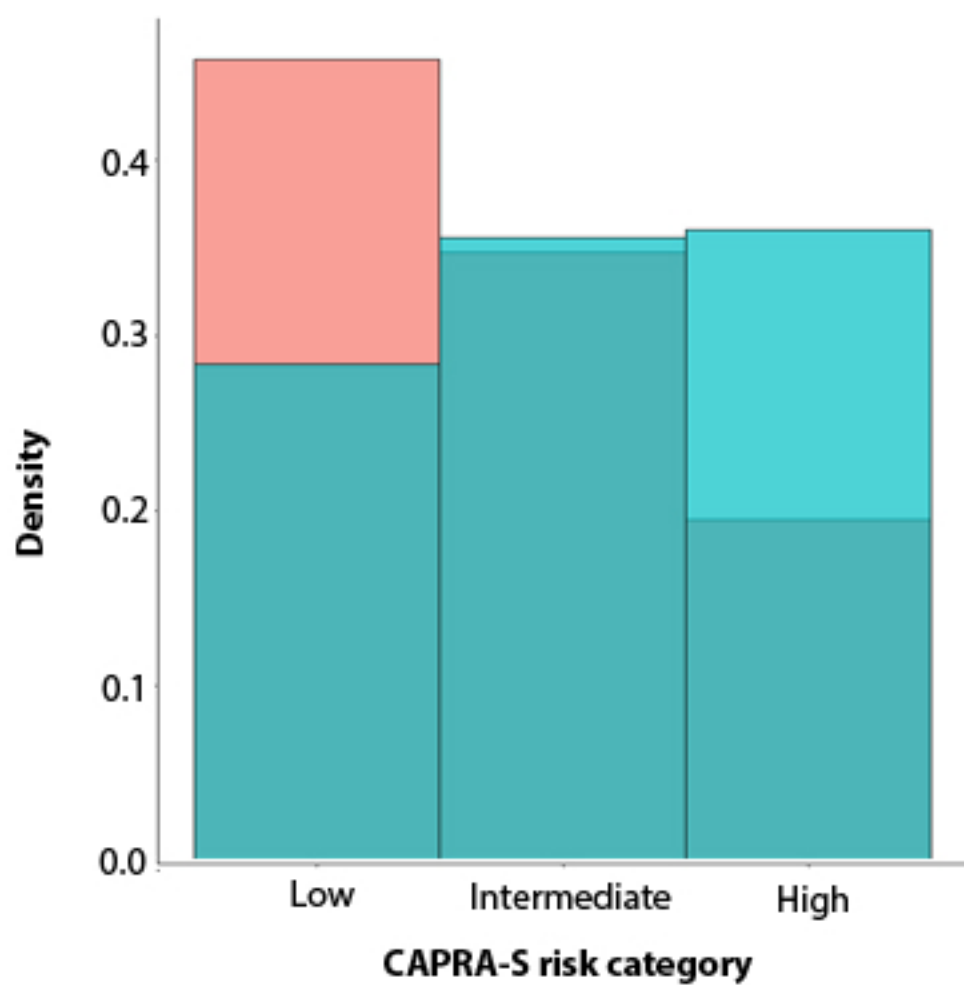

Supplement: Supplementary file 4 — Additional file 4: Supplementary Figure 4. Density histogram plots for 38G-positive and 38G-negative cases within the validation (TCGA) cohort, stratified by (A) continuous CAPRA-S risk score, or (B) CAPRA-S risk category, divided into low (0–2), intermediate (3–5), or high (6–12) risk groups. Overlaid density plots in (A) highlight the differences in peaks and distribution between gene model-selected and gene model-negative cases among the CAPRA-S risk scores. Figure generated using the R (v3.6.1) package ggplot2 (v3.2.1). [file 12885_2020_7438_MOESM4_ESM.pdf]

# CAPRA-S

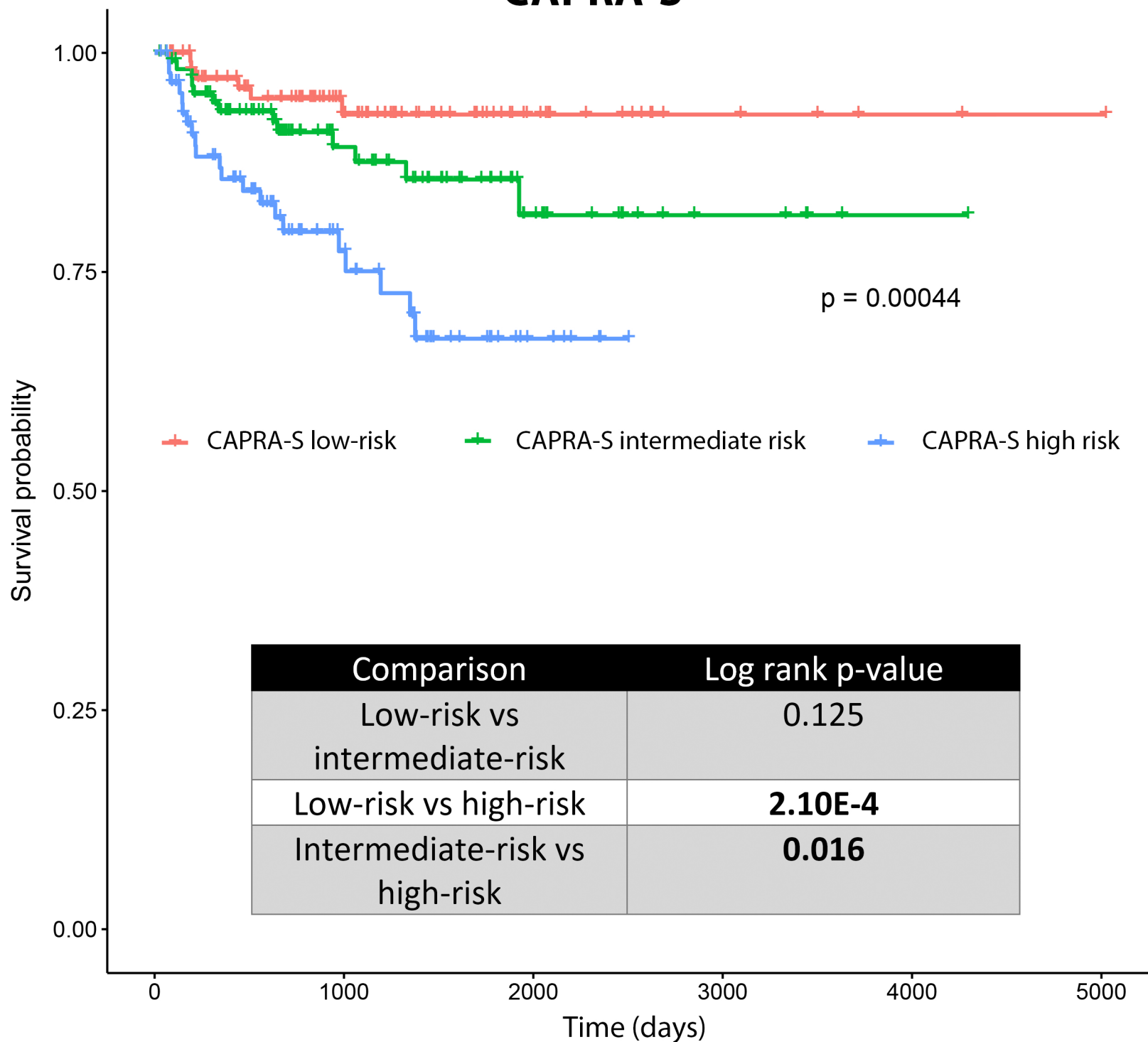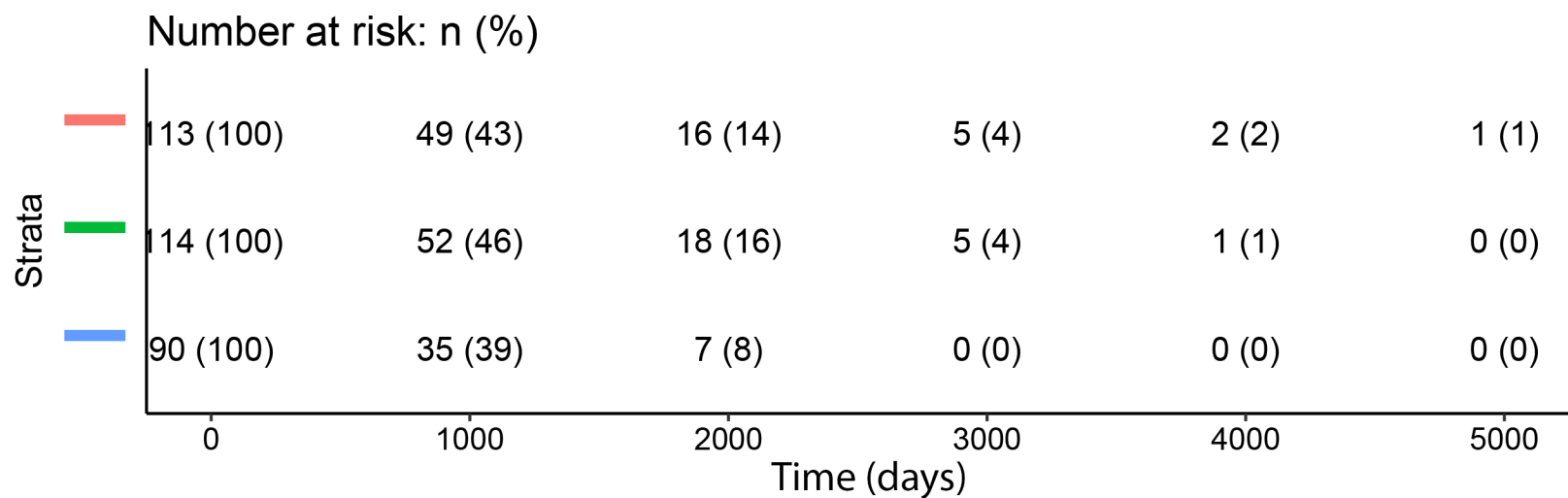

Supplement: Supplementary file 5 — Additional file 5: Supplementary Figure 5. Univariate Kaplan-Meier curve for prediction of BCR in the validation (TCGA) cohort. The three risk categories of CAPRA-S are assessed, with log-rank p-values for pairwise comparisons between risk categories indicated in the accompanying chart. Overall log-rank p-value is indicated on the graph. Below: Risk table indicating the number of patients in each group at risk at various timepoints. Figure generated using the R (v3.6.1) package survminer (v0.4.6). [file 12885_2020_7438_MOESM5_ESM.pdf]
