# Supplementary material for: A 38-gene model comprised of key TET2-associated genes shows additive utility to high-risk prostate cancer cases in the prognostication of biochemical recurrence
Source: BMC Cancer. 2020 Oct 2;20:953. doi: 10.1186/s12885-020-07438-4 (PMC7530956; doi:10.1186/s12885-020-07438-4)
Supplement: Supplementary file 7 — Additional file 7. Supplementary Methods. [file 12885_2020_7438_MOESM7_ESM.docx]

**Supplementary Methods**

*Gene set selection criteria*

A final gene set for use in random forest-based recursive feature elimination was chosen based on data from CRISPR-Cas9 generated *TET2-*knockout prostate cells and a low-*TET2-e*xpressing subset of tumors in the TCGA dataset in order to identify a set of genes exhibiting upregulation or downregulation associated with *TET2* loss in prostate tumors. Firstly, RNA sequencing of two such *TET2-*KO clones (CR1 and CR2), as well as parental normal prostate RWPE-1 cells, was performed as previously described by our group. [17] Differential expression analysis was performed using edgeR. 4192 genes were identified as exhibiting significantly decreased expression in either CR1 or CR2 as compared to parental RWPE-1 cells (p<0.05, FC<0.75), while 3819 genes exhibited significantly increased expression in *TET2-*KO cells (p<0.05, FC>1.5).

Of the 4192 downregulated genes, 780 genes exhibited significant loss of expression below the Bonferroni-corrected p-value threshold of 1.193E-5 (Mann-Whitney U test) in the low-*TET2* TCGA subset, where this subset was identified as tumors within the bottom 10^th^ percentile of *TET2* expression (n=43) as compared to normal prostate tissue from the same cohort (n=35). Similarly, 360 genes exhibited significant gain of expression (Bonferroni-corrected p<1.309E-5, Mann-Whitney U test) in this dataset. Uncharacterized genes were removed from this list, leaving a total of 1122 genes. This gene set was considered to be comprised of both upregulated and downregulated genes associated with *TET2,* and was used for downstream analysis.
